# Modelling effects of internalized antibody: a simple comparative study

**DOI:** 10.1186/1742-4682-11-11

**Published:** 2014-02-13

**Authors:** Vladas Skakauskas, Pranas Katauskis, Alex Skvortsov, Peter Gray

**Affiliations:** 1 Faculty of Mathematics and Informatics, Vilnius University, 24 Naugarduko st., LT-03225 Vilnius, Lithuania; 2 Defence Science and Technology Organisation, 506 Lorimer st., VIC 3207 Melbourne, Australia

**Keywords:** Toxin, Antibody, Cell receptor, Intracellular transport

## Abstract

**Background:**

The modelling framework is proposed to study protection properties of antibodies to neutralize the effects of the plant toxin (ricin). The present study extends our previous work by including (i) the model of intracellular transport of toxin to the Endoplasmic Reticulum and (ii) the model of the internalised antibodies (when antibody is delivered directly into the cytosol).

**Method:**

Simulation of the receptor-toxin-antibody interaction is implemented by solving the systems of PDEs (advection-diffusion models) or ODEs (rate models) for the underlying transport coupled with mass-action kinetics.

**Results:**

As the main application of the enhanced framework we present a comparative study of two kinds (external and internalised) of antibodies. This comparison is based on calculation of the non-dimensional protection factor using the same set of parameters (geometry, binding constants, initial concentrations of species, and total initial amount of the antibody).

**Conclusion:**

This research will provide a framework for consistent evaluation and comparison of different types of antibodies for toxicological applications.

## Background

The plant toxin ricin made from the seeds of the castor oil plant is highly toxic to mammalian cells. It is one of the deadliest toxins known and is classified as a potential bioterror agent for which no treatment is available. Some promising results have been shown recently in the immunotherapeutic approach, i.e. application of antibodies to neutralise the effects of ricin [1-4]. With the recent progress in bio-engineering, antibodies with high affinity have been generated. The development and production of new antibodies is still an expensive process that usually includes extensive experimental studies with continuous experimental refinement of antibodies properties. Evidently, that such a retrospective evaluation of different antibodies aiming at selection of the best candidate may become very time and resource consuming.

In order to reduce this experimental burden a simple (but scientifically consistent) modelling framework has been recently proposed [5-9]. This framework enables extensive theoretical optimization studies to increase the protective potential of antibodies before proceeding with targeted experimental studies.

The mechanism of ricin intracellular transport involves a number of steps each with its complex phenomenology which are well-documented (see [1-4] and Refs. therein). Ricin consists of an *A* (RTA) and *B* (RTB) chain linked by a disulphide bond. RTB binds to a cell surface receptor triggering uptake and retrograde transport to the Endoplasmic Reticulum (ER). In the ER, the RTA and RTB chains are separated and the RTA is translocated across the ER membrane into the cytosol. Subsequently RTA reaches ribosome and damages the protein production machinery of the cell resulting in the cell death. In this context, the toxin concentration in the cytoplasm near ER becomes the critical quantity to estimate the toxicological impact of ricin on the cell and evaluate the protective potential of the antibody. This was a motivation to introduce a consistent quantitative characteristic for antibody comparison (see below).

For the sake of parametrisation simplicity the coarse-grained modeling framework proposed in [5-9] seemingly ignores these fine details of toxin binding and internalisation. In fact, it is aimed at capturing the complexity of these processes by means of a small number of ‘aggregated’ rate constants that can be (or have been) evaluated experimentally or numerically. Such kind of models becomes a conventional tool in pharmacological modeling (for example, see [10] and Refs. therein). From the chemical point of view the framework is similar to one well-established in electrochemistry where it is used for estimation of uptake rates of the heavy metal ions from the environment, see [11-13]. A practical application of the proposed models involves a numerical (or sometimes analytical) solution of a nonlinear system of PDEs (diffusion kinetics) for a given set (or range) of antibody parameters (i.e. binding rates, concentration) to infer the effect of these parameters on the protective potential of the antibody.

**Figure 1:**
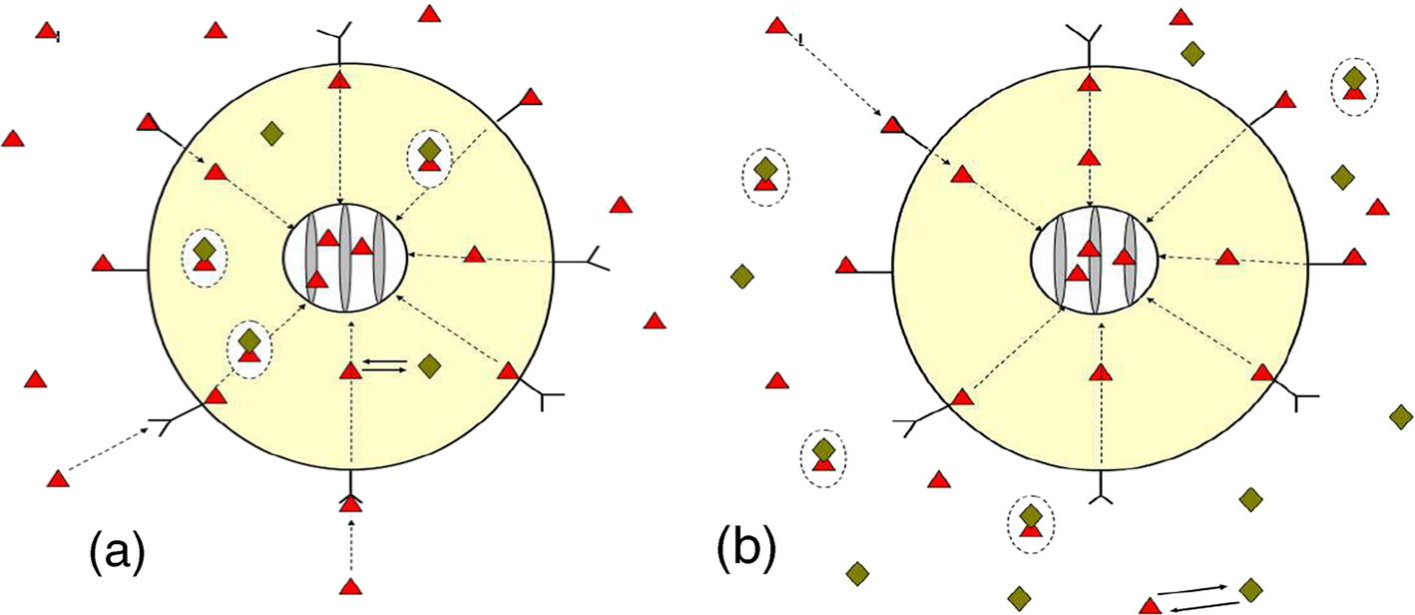
**The schematic diagram of receptor-toxin-antibody system: (a) - Scenario I and (b) - Scenario II.** △ – toxin, ◇ – antibody, conglutinated △ and ◇ in circle – toxin-antibody complex, external sphere – cell membrane, internal sphere – ER envelope.

In the present paper we extend our previous work [5-9] by refining models for intracellular transport and chemical interaction of species. Motivated by experimental studies available in the literature [1-4] and possible toxicological applications we consider two scenarios of antibody delivery. In the first scenario (below refer to as *Scenario I*) the antibody is placed inside the cell between the ER and cell membrane. The toxin initially is delivered outside the cell (in the extracellular domain). Then it moves toward the cell and interacts with receptors on the cell membrane. Some of toxin penetrates into the cell, where it further moves toward the ER (see Figure 1a) and eventually interacts with antibody.

In the second scenario (*Scenario II*) the antibody is delivered outside the cell where it initially interacts with the toxin partially neutralising it. The free toxin moves toward the cell membrane where it interacts with membrane receptors. Some of toxin penetrates into the cell where it is subsequently transported toward ER (see Figure 1b).

To model biologically complex phenomenology of species transport in cell we employ the following physical mechanisms: (i) diffusion (for species in extracellular space), (ii) diffusion and advection (direction transport via microtubule network) for toxin and only diffusion for antibody and toxin-antibody complex inside the cell, (iii) mass-action kinetics (to form complexes and describe binding processes).

Although all parts of the model have been implemented with the standard numerical algorithm (an implicit finite-difference scheme [14]), an interpretation of its output and parameters tuning requires some domain knowledge (solution of nonlinear systems of PDEs). In order to reduce dimension of the parameter space of the models and ameliorate their operational application we also considered so-called simplified (or reduced) models for each scenario. The simplified (sometimes called compartment) models are based on the ‘well-mixed’ assumption which states that all species have uniform concentration across the modeling domain. This assumption eliminates the a necessity to calculate the gradient driven fluxes in the models (i.e. diffusion) leading to a significant simplification (translation from PDEs to ODEs). By comparing outputs of complete and reduced models we can infer and defensively comment on the trade-off between simplicity and fidelity in modeling approach to an evaluation of each scenario.

As the main application of this framework we present a comparative study of external (conventional) and internalised antibodies. More specifically, for the same set of parameters (geometry, binding constants, initial concentration or initial amount of antibody) we calculate the non-dimensional parameter

(1) $$\delta (t)=\frac{u_{T}(t,\rho_{n}){|}_{A\in \Omega_{i}}}{u_{T}(t,\rho_{n}){|}_{A\in \Omega_{e}}},$$

which is a ratio of reductions of toxin concentration in the cytoplasm due to introduction of two kinds of antibodies. Here $u_{T}(t,\rho_{n}){|}_{A\in \Omega_{i}}$, $u_{T}(t,\rho_{n}){|}_{A\in \Omega_{e}}$ is the concentration of internalized toxin near ER for the case of internalized (*i*) and external antibody (*e*). The time evolution of this aggregated parameter *δ*(*t*) enables a consistent comparison of two scenarios of antibody treatment (i.e. either delivered extracellularly or into the cytosol). For instance, the condition *δ*<1 indicates that internalised antibody performs better than conventional antibody, while at *δ*>1 the conventional antibody outperforms. This comparison is the main outcome of our study.

The paper is organised as follows. In Section ‘The models’ we introduce models for toxin-antibody interaction outside and inside the cell. Results are discussed in Section ‘Numerical results’. Conclusions and summarising remarks are presented in Section ‘Conclusions’.

## Notation

We use the notation of papers [5-7]: 

*T* and *A* – the toxin and antibody;

*C*=*TA* – the toxin-antibody complex (nontoxic);

*ρ* – the distance to the origin;

*S*_*m*_={*ρ*:*ρ*=*ρ*_*m*_} – the surface of the membrane of a spherical cell;

*S*_*e*_={*ρ*:*ρ*=*ρ*_*e*_}, *ρ*_*e*_>*ρ*_*m*_ – the surface of the external sphere (external surface of *Ω*_*e*_);

*Ω*_*e*_={*ρ*:*ρ*∈(*ρ*_*m*_,*ρ*_*e*_)} – the extracellular domain;

*S*_*n*_={*ρ*:*ρ*=*ρ*_*n*_}, *ρ*_*n*_<*ρ*_*m*_ – the surface of the spherical envelope of the domain occupied by ER;

*Ω*_*i*_={*ρ*:*ρ*∈(*ρ*_*n*_,*ρ*_*m*_)} – the intracellular domain;

*r*_0_ – the concentration of receptors confined to the cell membrane;

*θ* – the fraction of the toxin-bound receptors;

*r*_0_*θ* – the concentration of the toxin-bound receptors;

*r*_0_(1-*θ*) – the concentration of the free receptors;

*u*_*A*_ and *u*_*C*_ – the concentrations of the antibody and toxin-antibody complex, respectively, in the domains *Ω*_*i*_ or *Ω*_*e*_;

*u*_*T*_ – the toxin concentration in *Ω*_*e*_∪*Ω*_*i*_;

$u_{T}^{0},$$u_{A}^{0e}$, $u_{A}^{0i}$ – the initial concentrations of the toxin and antibody in *Ω*_*e*_ and *Ω*_*i*_, respectively;

*κ*_*T*_, *κ*_*A*_, *κ*_*C*_ – the diffusivity of the toxin, antibody, and toxin-antibody complex, respectively;

*k*_1_, *k*_-1_ – the forward and reverse constants of the toxin-antibody reaction rate;

*k*_2_ and *k*_-2_ – the forward and reverse binding rate constants of the toxin and receptor confined on the membrane;

*k* – the toxin internalization rate constant from *Ω*_*e*_ across the membrane into the cell;

*γ* – the toxin absorption rate constant describing toxin influx into ER;

*v*(*ρ*), *v*>0 – the toxin advective velocity;

*∂*_*t*_=*∂*/*∂t*, $\Delta =\rho^{-2}\frac{\partial }{\partial \rho }(\rho^{2}\frac{\partial }{\partial \rho })$ – the Laplace operator.

## The models

As mentioned above we study two scenarios of antibody delivery. In *Scenario I* the antibody is delivered inside the cell, i.e. in domain *Ω*_*i*_ (space between the ER and cell membrane) while in *Scenario II* it is delivered outside the cell (space between cell membrane and external boundary of the extracellular domain, *Ω*_*e*_). In the *Scenario I* toxin being initially in *Ω*_*e*_ (extracellular domain) moves toward the cell and interacts with receptors on the cell membrane. Subsequently some of toxin penetrates into the intracellular domain, *Ω*_*i*_, where it moves toward ER and interacts with antibody. Toxin competitively reacts with antibody in the extracellular domain (*Scenario II*) or inside the cell (*Scenario I*). This ‘blocking’ reaction results in a reduced toxin concentration on the ER envelope and is the main bio-chemical mechanism of toxin neutralization by antibody.

In order to extend our results from a single cell model to many-cell systems we impose the no-toxin flux boundary condition on the external surface *S*_*e*_ of the extracellular domain *Ω*_*e*_ (to mimic periodicity of the infinite system). This also accounts for conservation of species in the system and enables consistent simulation of depletion effects (see [13] for details).

It is well-known that binding of toxin to antibody and toxin to receptors are reversible reactions, that can be described by the equations of mass-action kinetics (see [5-10] and Refs. therein). In the context of study of processes in the Scenario I these equations have been modified to include toxin directional transport and diffusion of all species in the intracellular domain (by adding appropriate advection and diffusion terms, e.g. see [15,16]). To describe extracellular transport and toxin binding by cell receptors we model the cell as a partially absorbing sphere with distributed binding sites that follow the Langmuir adsorption model. In the intracellular domain of the *Scenario I* the toxin transport is modelled by the advection-diffusion equation in which an advective velocity (drift) is introduced to account for directional transport via microtubule network. For the transport of the antibody and antibody-toxin complexes we still use the pure diffusion mechanism. We also assume that antibody and toxin-antibody complexes do not internalize and never cross the cell membrane or the ER envelope (internal concentric sphere). The schematic diagram of receptor-toxin-antibody system is presented in Figure 1.

Since the process of receptor binding is very rapid (seconds) and toxin internalization is relatively slow (hours) we can effectively decoupled the model for extracellular and intracellular domains and solve them sequentially. The relative values of time scales for receptor binding and toxin internalization have also been validated retrospectively and found to be consistent with the initial assumption of domains decoupling (see below).

The Advection-Diffusion Model used to evaluate *Scenario I* is an extension of the models previously published [4-9]. The following new processes have been included in our simulations: (i) the intracellular traffic that captures the diffusion of all species and the main features of the toxin microtubule transport, (ii) chemical interaction of all species inside the cell (see [15] and Refs. therein), and (iii) process of the toxin transport to ER.

In order to model toxin transport towards the ER a special boundary condition was introduced on the ER envelope. The radius of that spherical envelope was about the size of the domain occupied by the ER. It is assumed that the mixed (or radiation) boundary condition for species concentrations can capture (at least phenomenologically) the complexity of toxin kinetics inside the ER [17].

Now we can formulate the equations for the Advection-Diffusion Model (refer to as the ADM1 model). In extracellular space *Ω*_*e*_ we have the following set of equations:

(2) $$\left\{\begin{array}{l} \partial_{t}u_{T}=\kappa_{T}\Delta u_{T},\;\rho \in (\rho_{m},\rho_{e}),\;t>0,\\ \partial_{\rho }u_{T}=0,\;\rho =\rho_{e},\;t>0,\\ \partial_{\rho }u_{T}=\frac{r_{0}}{\kappa_{T}}(k_{2}(1-\theta )u_{T}-k_{-2}\theta ),\;\rho =\rho_{m},\;t>0,\\ u_{T}{|}_{t=0}=u_{T}^{0},\;\rho \in (\rho_{m},\rho_{e}), \end{array}\right)$$

(3) $$\left\{\begin{array}{l} \partial_{t}\theta =k_{2}(1-\theta )u_{T}-k_{-2}\theta -\mathrm{k\theta},\;\rho =\rho_{m},\;t>0,\\ \theta {|}_{t=0}=0,\;\rho =\rho_{m}. \end{array}\right)$$

Function *θ* determined from solution by these equations is used as the boundary condition in the equations in intracellular domain *Ω*_*i*_:

(4) $$\left\{\begin{array}{l} \partial_{t}u_{T}=-k_{1}u_{T}u_{A}+k_{-1}u_{C}+\kappa_{T}\Delta u_{T}+\partial_{\rho }({\text{vu}}_{T})+2{\text{vu}}_{T}/\rho ,\\ \;\rho \in (\rho_{n},\rho_{m}),\;t>0,\\ \kappa_{T}\partial_{\rho }u_{T}=r_{0}\mathrm{k\theta}-vu_{T},\;\rho =\rho_{m},\;t>0,\\ \kappa_{T}\partial_{\rho }u_{T}=(\gamma -v)u_{T},\;\rho =\rho_{n},\;t>0,\\ u_{T}{|}_{t=0}=0,\;\rho \in (\rho_{n},\rho_{m}), \end{array}\right)$$

(5) $$\left\{\begin{array}{l} \partial_{t}u_{A}=-k_{1}u_{T}u_{A}+k_{-1}u_{C}+\kappa_{A}\Delta u_{A},\;\rho \in (\rho_{n},\rho_{m}),\;t>0,\\ \partial_{\rho }u_{A}=0,\;\rho =\rho_{m},\;t>0,\\ \partial_{\rho }u_{A}=0,\;\rho =\rho_{n},\;t>0,\\ u_{A}{|}_{t=0}=u_{A}^{0i},\;\rho \in (\rho_{n},\rho_{m}), \end{array}\right)$$

(6) $$\left\{\begin{array}{l} \partial_{t}u_{C}=k_{1}u_{T}u_{A}-k_{-1}u_{C}+\kappa_{C}\Delta u_{C},\;\rho \in (\rho_{n},\rho_{m}),\;t>0,\\ \partial_{\rho }u_{C}=0,\;\rho =\rho_{m},\;t>0,\\ \partial_{\rho }u_{C}=0,\;\rho =\rho_{n},\;t>0,\\ u_{C}{|}_{t=0}=0,\;\rho \in (\rho_{n},\rho_{m}). \end{array}\right)$$

The set of Eqs. (2)–(6) composes the ADM1 model. The main parameter of interest for the *Scenario I* is the antibody protection factor (a relative reduction of the internalized toxin due to application of antibody) defined by the expression

(7) $$\mu_{1}(t)=\frac{u_{T}(t,\rho_{n};u_{A}^{0}){|}_{A\in \Omega_{i}}}{u_{T}(t,\rho_{n};0)}.$$

By definition, 0≤*μ*_1_(*t*)≤1 with the lower values of *μ*_1_(*t*) corresponding to the more profound therapeutic effect of antibody treatment.

To simplify the ADM1 model we employ the ‘well-mixed’ assumption [5]. This assumption implies that all species (toxin, antibody, and toxin-antibody complex) are uniformly distributed in the calculation domain for all time, so there is no spatial gradients of concentrations. Under this condition all flux terms disappear from the equations of ADM1. The process of toxin internalization (i.e. flux of toxin through the cell surface *S*_*m*_ and ER envelope *S*_*n*_) can be modelled as an appropriate rate of toxin removal from *Ω*_*e*_ and *Ω*_*i*_, respectively.

It is worth emphasizing that a reduction to the ‘well-mixed’ (or compartment) models requires fulfilment of some condition that can be formulated in terms of a smallness of the ratio *ε*=*τ*_*κ*_/*τ*_*R*_, where *τ*_*κ*_ and *τ*_*R*_ is a diffusion and reaction time scale in the system, respectively (diffusion can quickly restore any spatial inhomogeneity of species distribution maintaining uniform concentration). In the simplest case *τ*_*κ*_∼*a*^2^/*κ* and *τ*_*R*_∼1/*k*, where *a* is a characteristic length scale, *κ* is a scale of diffusivity and *k* is the scale of reaction rate. This is condition for what is called diffusion- or reaction–dominated regimes in diffusion kinetics [18]. Indeed, this condition does not always hold and we do not assume that it satisfies automatically in the context of our study. Since our models are characterized by a number of spatial scales (extracellular and intracellular domains, ER envelope), hierarchy of reaction rates and diffusion coefficients, it is very difficult (or even impossible) to formulate and validate any general criteria for the feasibility of the well-mixed assumption. For this reason we take a heuristic approach in which this assumption is validated retrospectively by comparing the output of the models, viz. the full (advection-diffusion) and reduced (compartment) models.

Under the well-mixed assumption the ADM1 model reduces to two ODEs.

(8) $$\left\{\begin{array}{l} {\dot{u}}_{T}=-k_{3}r_{0}(k_{2}(1-\theta )u_{T}-k_{-2}\theta ),\;t>0,\\ u_{T}{|}_{t=0}=u_{T}^{0},\\ \dot{\theta }=k_{2}(1-\theta )u_{T}-k_{-2}\theta -\mathrm{k\theta},\;t>0,\\ \theta {|}_{t=0}=0, \end{array}\right)$$

for extracellular domain *Ω*_*e*_ and three ODEs for inracelluar domain:

(9) $$\left\{\begin{array}{l} {\dot{u}}_{T}=-k_{1}u_{T}u_{A}+k_{-1}u_{C}+{\text{kk}}_{4}r_{0}\theta -k_{5}\gamma u_{T},\;\;t>0,\\ u_{T}{|}_{t=0}=0,\\ {\dot{u}}_{A}=-k_{1}u_{T}u_{A}+k_{-1}u_{C},\;t>0,\\ u_{A}{|}_{t=0}=u_{A}^{0i},\\ {\dot{u}}_{C}=k_{1}u_{T}u_{A}-k_{-1}u_{C},\;t>0,\\ u_{C}{|}_{t=0}=0, \end{array}\right)$$

with $k_{3}=3\rho_{m}^{2}/(\rho_{e}^{3}-\rho_{m}^{3})$, $k_{4}=3\rho_{m}^{2}/(\rho_{m}^{3}-\rho_{n}^{3})$, $k_{5}=3\rho_{n}^{2}/(\rho_{m}^{3}-\rho_{n}^{3})$.

We call systems (8), (9) as the Well-Mixed Model 1 (WM1).

Analogously we formulate two models used to evaluate antibody protection properties in *Scenario II*. For the extracellular domain *Ω*_*e*_ we employ the Diffusion Model proposed in [5-7]:

(10) $$\left\{\begin{array}{l} \partial_{t}u_{T}=-k_{1}u_{T}u_{A}+k_{-1}u_{C}+\kappa_{T}\Delta u_{T},\;\;\rho \in (\rho_{m},\rho_{e}),\;t>0,\\ \partial_{\rho }u_{T}=0,\;\rho =\rho_{e},\;t>0,\\ \kappa_{T}\partial_{\rho }u_{T}=r_{0}\{k_{2}(1-\theta )u_{T}-k_{-2}\theta \},\;\rho =\rho_{m},\;t>0,\\ u_{T}{|}_{t=0}=u_{T}^{0},\;\rho \in (\rho_{m},\rho_{e}), \end{array}\right)$$

(11) $$\left\{\begin{array}{l} \partial_{t}u_{A}=-k_{1}u_{T}u_{A}+k_{-1}u_{C}+\kappa_{A}\Delta u_{A},\;\rho \in (\rho_{m},\rho_{e}),\;t>0,\\ \partial_{\rho }u_{A}=0,\;\rho =\rho_{e},\;t>0,\\ \partial_{\rho }u_{A}=0,\;\rho =\rho_{m},\;t>0,\\ u_{A}{|}_{t=0}=u_{A}^{0e},\;\rho \in (\rho_{m},\rho_{e}), \end{array}\right)$$

(12) $$\left\{\begin{array}{l} \partial_{t}u_{C}=k_{1}u_{T}u_{A}-k_{-1}u_{C}+\kappa_{C}\Delta u_{C},\;\rho \in (\rho_{m},\rho_{e}),\;t>0,\\ \partial_{\rho }u_{C}=0,\;\rho =\rho_{e},\;t>0,\\ \partial_{\rho }u_{C}=0,\;\rho =\rho_{m},\;t>0,\\ u_{C}{|}_{t=0}=0,\;\rho \in (\rho_{m},\rho_{e}), \end{array}\right)$$

(13) $$\left\{\begin{array}{l} \partial_{t}\theta =k_{2}(1-\theta )u_{T}-k_{-2}\theta -\mathrm{k\theta},\;\rho =\rho_{m},\;t>0,\\ \theta {|}_{t=0}=0,\;\rho =\rho_{m}, \end{array}\right)$$

with toxin concentration in *Ω*_*i*_ being described by the Advection-Diffusion equation

(14) $$\left\{\begin{array}{l} \partial_{t}u_{T}=\kappa_{T}\Delta u_{T}+\partial_{\rho }({\text{vu}}_{T})+2{\text{vu}}_{T}/\rho ,\;\rho \in (\rho_{n},\rho_{m}),\;t>0,\\ \kappa_{T}\partial_{\rho }u_{T}=r_{0}\mathrm{k\theta}-vu_{T},\;\rho =\rho_{m},\;t>0,\\ \kappa_{T}\partial_{\rho }u_{T}=(\gamma -v)u_{T},\;\rho =\rho_{n},\;t>0,\\ u_{T}{|}_{t=0}=0,\;\rho \in (\rho_{n},\rho_{m}). \end{array}\right)$$

We refer to systems (10), (13) as the Advection-Diffusion Model 2 (ADM2). The main parameter of interest evaluated with this model is the antibody protection factor

(15) $$\mu_{2}(t)=\frac{u_{T}(t,\rho_{n};u_{A}^{0}){|}_{A\in \Omega_{e}}}{u_{T}(t,\rho_{n};0)}.$$

Similarly to the *Scenario I* we can apply the well-mixed assumption to simplify this model and reduce it to a set of ODEs

(16) $$\left\{\begin{array}{l} {\dot{u}}_{T}=-k_{1}u_{T}u_{A}+k_{-1}u_{C}-k_{3}r_{0}(k_{2}(1-\theta )u_{T}-k_{-2}\theta ),\;\;t>0,\\ u_{T}{|}_{t=0}=u_{T}^{0},\\ {\dot{u}}_{A}=-k_{1}u_{T}u_{A}+k_{-1}u_{C},\;t>0,\\ u_{A}{|}_{t=0}=u_{A}^{0e},\\ {\dot{u}}_{C}=k_{1}u_{T}u_{A}-k_{-1}u_{C},\;t>0,\\ u_{C}{|}_{t=0}=0,\\ \dot{\theta }=k_{2}(1-\theta )u_{T}-k_{-2}\theta -\mathrm{k\theta},\;t>0,\\ \theta {|}_{t=0}=0 \end{array}\right)$$

in *Ω*_*e*_ and

(17) $$\left\{\begin{array}{l} {\dot{u}}_{T}=kk_{4}r_{0}\theta -\gamma k_{5}u_{T},\;t>0,\\ u_{T}{|}_{t=0}=0 \end{array}\right)$$

in *Ω*_*i*_. We call system (16) and (17) as the Well-Mixed Model 2 (WM2).

To compare the antibody protection capability in both scenarios (i.e. to infer which scenario of antibody delivery is more beneficial for a particular type of antibody) we use function *δ*(*t*) defined in Eq. (1) in which $u_{T}(t,\rho_{n}){|}_{A\in \Omega_{i}}$ and $u_{T}(t,\rho_{n}){|}_{A\in \Omega_{e}}$ are the toxin concentrations near ER determined for the *Scenario I* and *II*, respectively. It is evident that the following identity holds, *δ*(*t*)=*μ*_1_(*t*)/*μ*_2_(*t*).

It is convenient to translate Eqs. (1)–(17) to the non-dimensional form by using scales of *τ*_∗_ (time), *l* (length), and *u*_∗_ (concentration). By substituting new variables, $\rho =l\bar{\rho },$$t=\tau_{*}\bar{t},$$r_{0}={\text{lu}}_{*}{\bar{r}}_{0}$, $u_{T}=u_{*}{ū}_{T},$$u_{A}=u_{*}{ū}_{A},$$u_{C}=u_{*}{ū}_{C},$$u_{T}^{0}=u_{*}{ū}_{T}^{0},$$u_{A}^{0}=u_{*}{ū}_{A}^{0}$, ${\bar{k}}_{1}=\tau_{*}u_{*}k_{1},$${\bar{k}}_{2}=\tau_{*}u_{*}k_{2},$${\bar{k}}_{-1}=\tau_{*}k_{-1},$${\bar{k}}_{-2}=\tau_{*}k_{-2},$$\bar{k}=\tau_{*}k$, ${\bar{\kappa }}_{T}=\tau_{*}\kappa_{T}l^{-2},$$\gamma =l\tau_{*}^{-1}\bar{\gamma },$$v=l\tau_{*}^{-1}\bar{v},$${\bar{\kappa }}_{A}=\tau_{*}\kappa_{A}l^{-2},$${\bar{\kappa }}_{C}=\tau_{*}\kappa_{C}l^{-2}$, ${\bar{k}}_{3}={\text{lk}}_{3},\;{\bar{k}}_{4}=lk_{4},\;{\bar{k}}_{5}=lk_{5}$, into (1)–(17) we can deduce the same systems, but now in non-dimensional form. Therefore, for simplicity in what follows, we treat system (1)–(17) as non-dimensional.

## Numerical results

We treated Eqs. (2)–(6), (8), (9), (10)–(14), and (16), (17) numerically for *t*>0 in spherically symmetric domains *ρ*∈(*ρ*_*m*_,*ρ*_*e*_) and *ρ*∈(*ρ*_*n*_,*ρ*_*m*_), respectively. We solve PDEs, Eqs. (2)–(6) and Eqs. (10)–(14), by an implicit finite-difference scheme [14]. To solve ODEs, Eqs. (8), (9) and Eqs. (16), (17), we apply the Runge–Kutta scheme.

Our selection of the values of parameters for the models (2)–(6), (8), (9), (10)–(14), and (16), (17) was motivated by the values available in the literature [2,5,19-21] with an extended range to allow exploration and illustration of the various transport regimes that are possible inside the cell. The following values were used in simulations [18]: *u*_∗_=6.02·10^13^ cm^-3^, *τ*_∗_=1 s, *r*_0_=1.6·10^4^/*S*_*m*_, where 1.6·10^4^ is the total number of receptors of the cell, *l*=10^-2^ cm,$S_{m}=4\pi \rho_{m}^{2}=4\pi \cdot 10^{-6}\;{\text{cm}}^{2}$, ${\bar{r}}_{0}=2.115\cdot 10^{-3}$. Values of the dimensionless parameters were the following: *ρ*_*n*_=0.02, *k*=3.3·10^-5^, *k*_1_=1.3·10^-2^, *k*_-1_=1.4·10^-4^, *k*_2_=1.25·10^-2^, *k*_-2_=5.2·10^-2^, *κ*_*A*_=10^-2^, *κ*_*C*_=10^-2^, $u_{T}^{0i}=0$, *r*_0_=0.21·10^-2^, *ρ*_*m*_=0.1, *ρ*_*e*_=0.18. Values of *κ*_*T*_ and *γ* are given in the legends of plots. The standard value of *v* is 0.001. Otherwise it is given in legends of plots.

We expect that the chosen values of the parameters were representative enough to illustrate a rich variety of possible scenarios of the evolution of the Receptor-Toxin-Antibody system and provide a reasonable estimate of time scales of the associated dynamics.

The Advection-Diffusion Models enables the fine spatial-temporal resolution of species concentration and provide valuable insights into the phenomenology of species interaction in our system. We found them to be useful tools for understanding the evolution of aggregated parameters *μ*_1_, *μ*_2_, *δ* predicted by our simulations. More specifically, by means of the Advection-Diffusion Models we were able to identify a number of important regimes of toxin penetration that may have significant implications for the assessment for the antibody protection properties.

These regimes can be best described by using the ‘transport phenomena’ terminology which is well-established in chemical engineering, for instance see [22,23]. Initially all species are concentrated in spherical layers between the external sphere, cell membrane, and ER envelope (i.e. (*ρ*_*m*_,*ρ*_*e*_) and (*ρ*_*n*_,*ρ*_*m*_)). This layer structure results in strong radial gradient of toxin concentration which facilitates the development of diffusion fluxes across the system (since cell membrane and ER envelope are ‘penetrable’). Without antibody toxin would eventually be flushed out of the system when it reaches the ER envelope driven by the pure diffusion mechanism. The advection velocity will change toxin transport at one part of the system (from diffusion dominated to advection dominated). How much toxin can be blocked by antibody during this transition to ER depends on a number of factors. They include the initial reactants concentration (toxin and antibody), relative value of toxin advection flux, and reaction time of toxin-antibody binding. It also depends on the availability of antibody in the areas of high toxin concentration to maximize the effective toxin-antibody binding. The last condition (local availability of antibody for binding toxin) is determined by the antibody diffusivity and can be validated by inspection of the solutions of Advection-Diffusion Models (spatial-temporal outputs). This validation enables justifiable comments on the relative reduction of toxin in the output of the system (on ER envelope) and the specific values of antibody protection parameters *μ*_1_, *μ*_2_. This approach was employed in our study to identify and investigate the most ‘vulnerable’ regimes of toxin penetration and comment on the shapes of functions *μ*_1_(*t*), *μ*_2_(*t*). Some of these regimes are presented in examples below.

Furthermore such a ‘chemical engendering’ approach provides a clear path for the system optimization. Indeed, from the formal point of view our system (cell) can be cast as a chemical reactor with the only output (product), being the toxin concentration at the ER envelope. In this context, the aim of antibody treatment is to reduce this output by distributing the minimal amount of a given antibody across the system. We anticipate that in such settings the optimization problem can be tacked in reduced parameter space by using the established framework for chemical reactor design [23,24].

**Figure 2:**
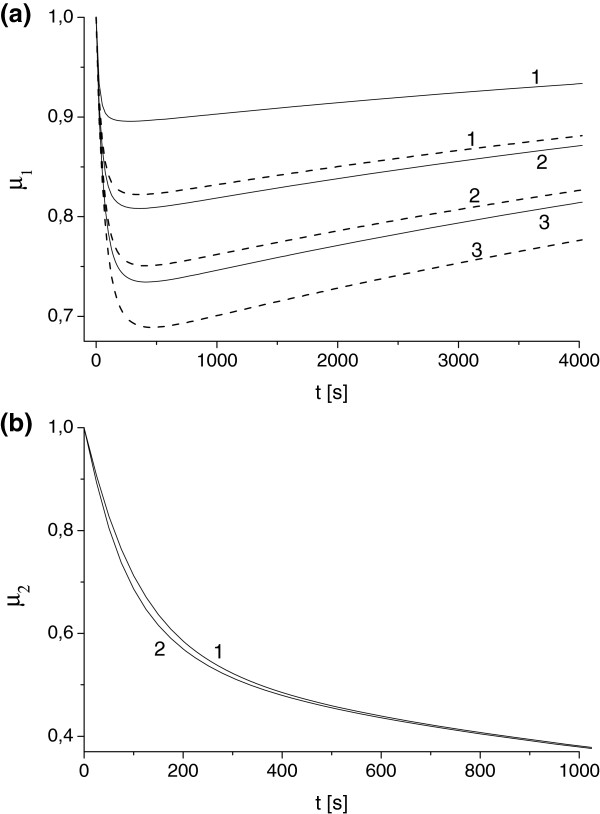
**Influence of toxin diffusivity and absorption constant on antibody protection factor. (a)** Scenario I. Solid and dashed lines correspond to value of *u*_*T*_ determined for *γ*=0.1 and *γ*=0.05. Toxin diffusivity *κ*_*T*_: 10^-2^ (1), 10^-3^ (2), 5·10^-4^ (3). **(b)** Scenario II. Absorption constant *γ*=0.1, toxin diffusivity *κ*_*T*_: 10^-2^ (1), 5·10^-4^ (2). $u_{A}^{0,e}=u_{A}^{0,i}=1$ in cases **(a)** and **(b)**.

**Figure 3:**
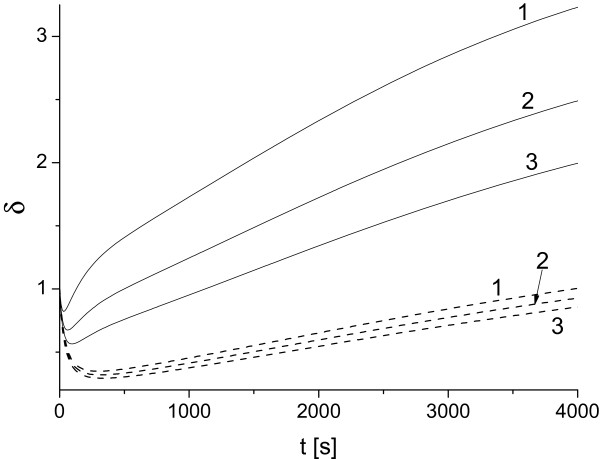
**Effect of variation of toxin diffusivity on parameter *δ* in case**$u_{A}^{0,e}=1$**, **$u_{A}^{0,i}=4.87$**.** Solid and dashed lines correspond to value of *δ* determined for *γ*=0.1 and *γ*=0.01. Toxin diffusivity *κ*_*T*_: 10^-2^ (1), 10^-3^ (2), 5·10^-4^ (3).

**Figure 4:**
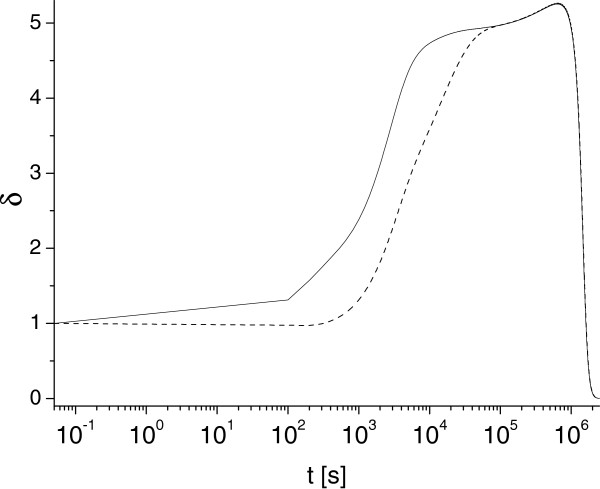
**Long-time asymptotic behavior of metric parameter *δ*(*t*).** Solid and dashed lines correspond to *δ* determined for *γ*=0.1 and *γ*=0.01 in case $u_{A}^{0,e}=u_{A}^{0,i}=1$, *κ*_*T*_=0.01.

**Figure 5:**
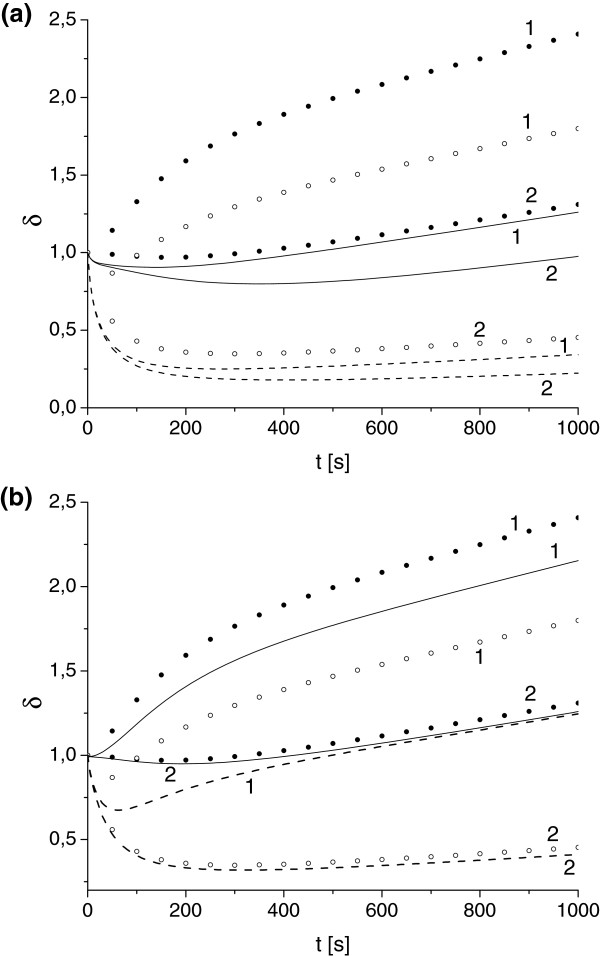
**Effect of variation of toxin diffusivity and absorption constant on parameter *δ*.** Toxin diffusivity: **(a)***κ*_*T*_=10^-4^, **(b)***κ*_*T*_=10^-3^. Solid ($u_{A}^{0,e}=u_{A}^{0,i}=1$) and dashed ($u_{A}^{0,e}=1$, $u_{A}^{0,i}=4.87$) lines correspond to *δ* determined by advection-diffusion models, and bullets ($u_{A}^{0,e}=u_{A}^{0,i}=1$) and circles ($u_{A}^{0,e}=1$, $u_{A}^{0,i}=4.87$) to *δ* determined by well-mixed models for *γ*: 0.1 (1), 0.01 (2).

**Figure 6:**
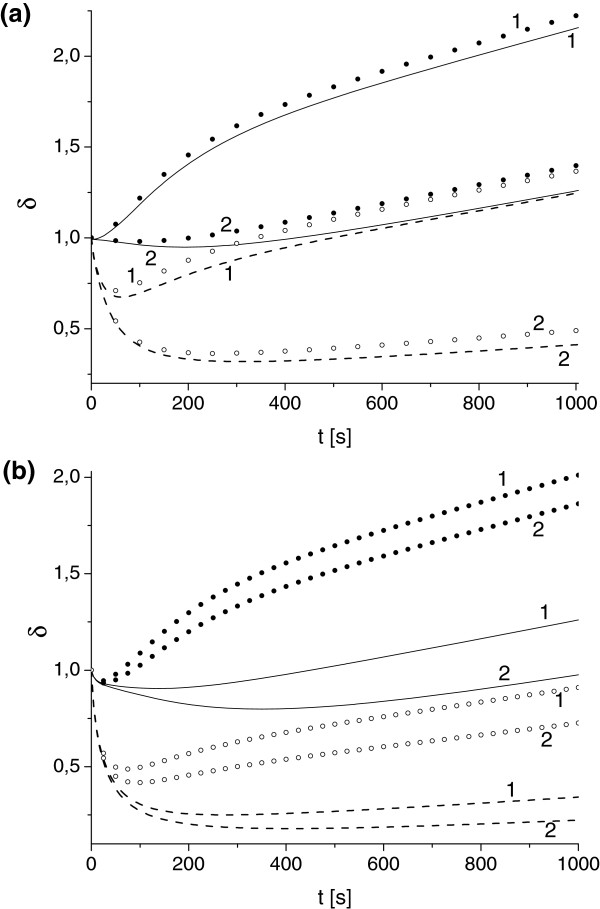
**Effect of variation of toxin advection velocity *v* on parameter*δ*.** Advection-diffusion models (solid lines): *v*=0.001, *γ*=0.01 (1), *v*=0.001, *γ*=0.1 (2), *v*=0.05, *γ*=0.01 (3), *v*=0.05, *γ*=0.1 (4); toxin diffusivity: **(a)***κ*_*T*_=10^-3^, **(b)***κ*_*T*_=10^-4^. Well-mixed models (dashed lines): *γ*=0.01 (1), *γ*=0.1 (2).

The results of the numerical solutions are presented in Figures 2, 3, 4, 5 and 6. The main purpose of our study was to estimate the effects of the toxin diffusivity, advective velocity, and the ‘ER interface’ parameter *γ* (rate constant of ER absorption) on the protective properties of the antibody in both scenarios. As such, most plots illustrate the effect of these parameters. In order to provide a consistent comparison of the simulation outputs we apply the same initial concentration of antibody or the same initial amount (but different concentration) of antibody. The equality of the initial amount leads to the following relation for the initial concentration

(18) $$u_{A}^{0i}=\eta u_{A}^{0e},\;\eta =\frac{{\left(\rho_{e}/\rho_{m}\right)}^{3}-1}{1-{\left(\rho_{n}/\rho_{m}\right)}^{3}}$$

which follows from the simple geometrical arguments. All calculations were performed for *ρ*_*e*_=0.18, *ρ*_*m*_=0.1, *ρ*_*n*_=0.02 for which *η*=4.871. This condition becomes especially important for the comparison of full and reduced models. Unless stated otherwise the value of all parameters (except depicted in plots) are assumed to be fixed and equal to those given above.

As mentioned, our simulations reveal a number of interesting regimes of toxin propagation. In particular, in *Scenario I* we observed that there was a range of parameters (*γ*, *κ*_*T*_, *v*) for which the diffusive flux of toxin, $\kappa_{T}\partial_{\rho }u_{T}{|}_{\rho =\rho_{m}}=(r_{0}\mathrm{k\theta}-vu_{T}){|}_{\rho =\rho_{m}}$, becomes negative near the cell membrane, i.e., it is opposite to the advective flux directed toward ER. This may reduce the total toxin flux inside the cell and provide more favorable condition for toxin-antibody binding.

In other words this phenomenon is a direct result of toxin transport being dominated by directional advection (due to velocity *v*). In our simulations this regime occurred for slow toxin transport characterized by parameters *γ*, *κ*_*T*_, *v* and the slow forward reaction rate for toxin-antibody binding. It is worth mentioning that the similar phenomenon has been reported in toxicological studies [17]. We found that the development of this ‘advection dominated’ region of toxin transport with reverse diffusive flux has a positive effect on antibody protection properties since it effectively increases the efficiency toxin-antibody binding (by increasing time available for toxin-antibody reaction). We also solved problem (2)–(9) using position-dependent advective velocity *v*(*ρ*) which is zero at the membrane and then rapidly tends to a constant value as *ρ*→*ρ*_*n*_. In this case because of the boundary condition (4)_2_, the diffusive toxin flux near the cell membrane is directed to ER.

Figure 2a illustrates solution for antibody protection factor for different values of toxin diffusivity *κ*_*T*_ and the ER absorption rate *γ* (with all other parameters being fixed). These results demonstrate the general trend of *δ*(*t*) increase with the increase of *κ*_*T*_ and *γ*. For function *μ*_1_(*t*) (*Scenario I*, Eq. (7)) we found noticeable non-monotonic behavior for all *κ*_*T*_ and *γ*. This implies that there is a time interval for which the protection potential of antibody in *Scenario I* reaches its maximum and then decays. According to Figure 2a this time interval is of order of 500 s.

Figure 2b demonstrates the evolution of antibody protection factor *μ*_2_(*t*) (*Scenario II*, Eq. (15)) for a range of parameters *κ*_*T*_ and *γ*. Although the shape of function *μ*_2_ is very different (it is always monotonically decreasing) we can conclude that the value of *μ*_2_ drops twice during the first time interval (about 500 s). Simulations for *γ*=0.1, 0.05 and *κ*_*T*_=10^-2^, 10^-3^, 10^-4^ provide almost identical outputs. This leads to an important conclusion of insignificance of parameters *κ*_*T*_ and *γ* for the prediction of antibody protection factors in *Scenario II* which is a direct consequence of equation decoupling for extracellular and intracellular domain discussed above.

The plots in Figure 3 illustrate the effect of variation of parameters *κ*_*T*_ and *γ* on the comparative protective skills of the same antibody. This effect is described in terms of metric factor *δ*(*t*), Eq. (1). Our simulations generally demonstrate the decrease of parameter *δ*(*t*) as *γ* or *κ*_*T*_ decrease. The simulations have been performed for the same initial amount of antibody (Eq. (18)) as well as for the same initial concentration (results not presented here). We observe that depending on value of *κ*_*T*_ and *γ* there exists a time interval in which *δ*(*t*)<1, i.e. the application of the antibody in *Scenario I* is more efficient than in *Scenario II*. We found that this interval grows as *κ*_*T*_ or *γ* decreases. Overall we found that the condition *δ*(*t*)<1 is rather sensitive to the ER absorption rate *γ*. This conclusion is intuitively clear since by reducing value of *γ* we also reduce the toxin flux through the system facilitating more effective antibody-toxin binding which, in turn, leads to more favorable output for *Scenario I*.

Figure 4 demonstrates some universal behaviors of function *δ*(*t*) that we identified for a broad range parameters of our study. Our simulations reveal that for some intermediate time (*t*∈(2·10^4^;10^6^) seconds) function *δ*(*t*) may approximately saturates to a constant plateau (depending on values of *γ* and *κ*), before it rapidly decays to zero. This is illustrated by two plots presented in Figure 4 (note different time scales). In particular we found that for $u_{A}^{0i}=u_{A}^{0e}$, *γ*=10^-1^, *κ*_*T*_=10^-2^ function *δ*(*t*) varies very insignificantly (less that 10%, between 4.9 and 5.2) for the time interval (*t*∈ [ 2·10^4^;5·10^5^]) seconds, then it decreases and vanishes as *t*→*∞*. Note, the limit *δ*(*t*)→0 occurs at the unpractically long times, so it has more methodologically interest. We do not have a clear explanation of the specific numerical value of this saturation limit (*δ*(*t*)≈5); this will require further investigation.

Figure 5 shows the comparative results of the performance of the full Advection-Diffusion models (ADM1, ADM2) and the reduced models (WM1, WM2). We compare estimations of the metric parameter *δ*(*t*) provided by these models for different toxin diffusivity *κ*_*T*_, ER absorbtion rate *γ*, and initial antibody concentration. We found that for the high toxin diffusivity (*κ*_*T*_≥10^-2^) the outputs of full model and reduced models are almost identical. The differences between the estimations of *δ*(*t*) increases as *κ*_*T*_ decreases and for *κ*_*T*_<10^-4^ the reduced models fails to provide any meaningful predictions (Figure 5b). We found that even for *κ*_*T*_>10^-4^ the reduced models usually poorly handle the non-monotonic behavior of function *δ*(*t*) at short times. At the long times we observed that for the broad range of parameters the full and reduced models are reasonably aligned and can yield reasonable estimation of *δ* as *t*→*∞* (within 20% accuracy), Figure 5a.

The plots in Figure 6 illustrate the effect of the toxin advective velocity, *v*, on the prediction of the protection factor *δ*(*t*), Eq. (1). Two different values of toxin advective velocity are used for simulations, *v*=10^-3^ and *v*=5·10^-4^ with *γ*=0.1. The toxin diffusivity was *κ*_*T*_=10^-3^ (Figure 6a) and 10^-4^ (Figure 6b) and the same values of the initial antibody concentration in both scenarios were used. Only two plots in these figures correspond to the well-mixed models, since they do not involve advective velocity *v*. In general we observed the growth of *δ*(*t*) as advective velocity increases, so *Scenario II* becomes more favorable. This is intuitively clear because with an increase of *v* the toxin neutralization time decreases and therefore *Scenario I* becomes less favorable. We also noticed the improved accuracy of reduced models as *v* increased. So, for given *κ*_*T*_ and *γ*, the difference between *δ*(*t*) determined by Advection-Diffusion models and Well-Mixed ones is small only if advective velocity, *v*, is small. But, for small *γ*, it dramatically increases as *v* grows, while for large *γ* it increases but not so dramatically. This is due to the switching to the advection dominated transport of toxin in the system.

## Conclusions

We present a new modeling framework to evaluate the protective potential of various antibodies. This framework is based on the equation for the diffusion- and advection-diffusion transport of all species in the extracellular and intracellular domains and include mass-action kinetics for toxin, receptor and antibody. The advection term and radiation boundary condition on the Endoplasmic Reticulum envelope were used to model the toxin transport via the microtubule network. We estimated the protection factor of antibody (relative reduction of toxin concentration near the ER) for two scenarios of operational relevance, viz., when the antibody is delivered externally to the cell and when it is delivered directly into the cytosol. To provide a consistent comparison of antibody performance we estimated the evolution of the metric factor *δ*(*t*), which is defined as the ratio of the protections factors of an antibody for each scenarios. Based on this definition the condition *δ*(*t*)<1 over a time span implies that the internally introduced antibody has better protective capability during this time while in the opposite case (*δ*(*t*)>1) the internalized antibody is more effective. In this study simulated both cases. Our models reveal that depending on values of some parameters (primary toxin diffusivity and ER absorbtion rate) the plot of function *δ*(*t*) can significantly change its shape while keeping the same short- and long-time limits, by undergoing a saturation stage at the intermediate times with a universal value of *δ*≈5.

To reduce the computational burden of the Advection-Diffusion Model (a system of nonlinear PDEs) we investigated its possible simplification. By employing the well-mixed assumption we reduced the Advection-Diffusion Model to the compartment or rate model (set of ODEs) which can be easily solved with any numerical solver. For an extended range of parameters we found that predictions of the compartment model can provide about ±20% accuracy in estimation of antibody protection potential (depending on values of some other parameters), but with decreasing toxin diffusivity limit the compartment model become a poor predictor and should not be used in this context. This finding can provide some appreciation of a possible trade-off between simplification and fidelity in toxicological modelling and can be an important criterion for selection of operational models.

We would like to emphasise that the comparative study reported in the present paper can be treated only as the first step for the application of the proposed modelling framework to the practical pharmacological studies. More extensive simulations (to provide statistically viable outcomes), data fitting and an established strategy for parameter optimization using toxicological observations will be necessary.

## Competing interests

The authors declare that they have no competing interests.

## Authors’ contributions

The work presented here was carried out in collaboration between ors. VS extended previous work by including the model of intracellular transport via diffusion and advection. PK developed the computation algorithms and carried out simulations. AS co-designed the model and validated its outcome against available analytical results. PG conducted a literature review, defined the research theme and interpreted the results. All authors contributed to the writing of the article and read and approve the final manuscript.
